# Supplementary material for: Computational and experimental insights into the chemosensory navigation of Aedes aegypti mosquito larvae
Source: Proc Biol Sci. 2019 Nov 20;286(1915):20191495. doi: 10.1098/rspb.2019.1495 (PMC6892055; doi:10.1098/rspb.2019.1495)
Supplement: Supplemental Information [file rspb20191495supp1.pdf]

Supplementary Materials

Supplementary Figures

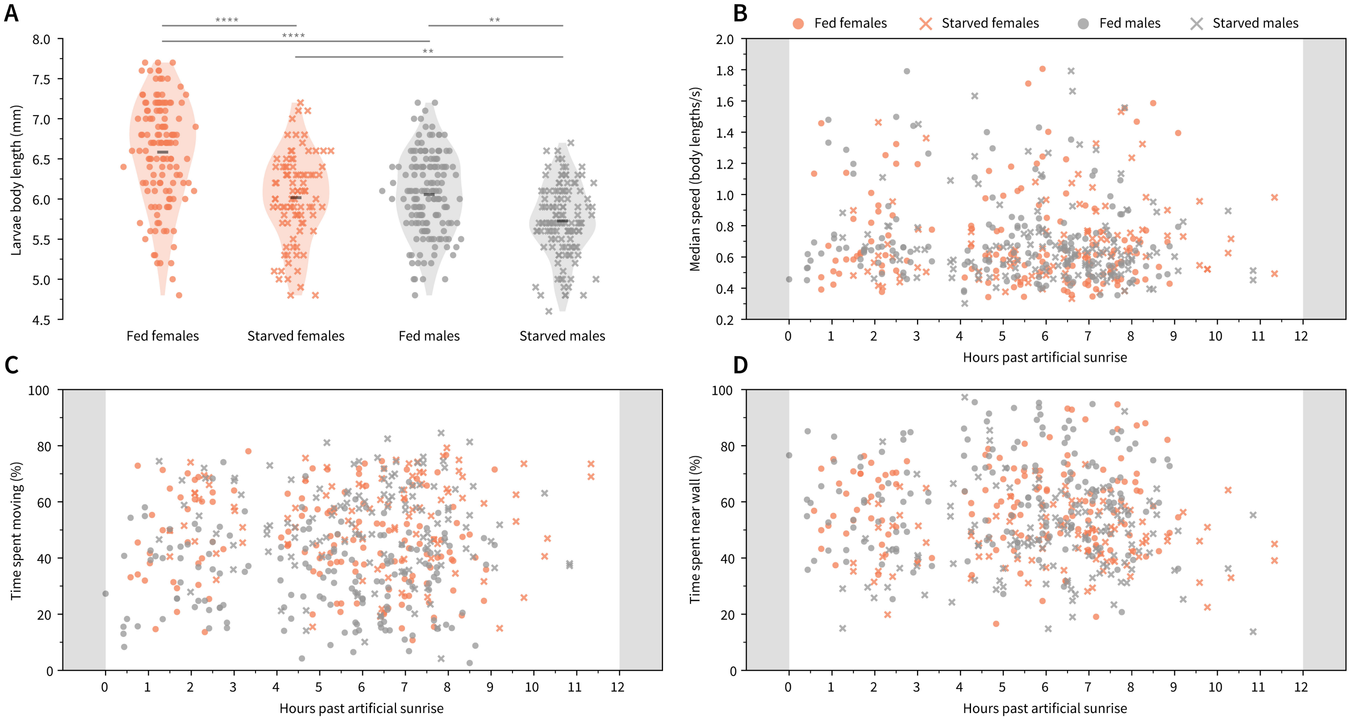

Figure S1: Effects of sex, physiological state, and circadian timing on larval physiology. A-D: Fed females (orange dots, n=135) and males (grey dots, n=153), starved females (orange X markers, n=89) and males (grey X markers, n=122). **A:** Violin plot. Scatter points show the body length (mm) for each individual, and the black bar is the mean across all individuals; asterisks denote significance values (Welch's t-test). Larval body length is influenced by sex and starvation state. **B:** No change was observed in median speed (body lengths/s) as a function of circadian timing. Note that the sampling rate throughout the day was not consistent due to the work schedule of experimenters involved in the project. **C:** No change was observed in time spent moving throughout daylight hours. **D:** No change was observed in proportion of time spent within one body length of the wall throughout daylight hours. **A-D:** For all effects shown above, we pooled measurements of all animals from the acclimation period. Our experiment results for each individual stimulus have far fewer animals for each sex, and we did not have sufficient power to analyze stimulus-specific sex differences. Nevertheless, we accounted for possible sex-specific confounds such as larval size or movement speed by normalizing the stimulus response of each animal to its activity during the pre-experiment acclimation period. In addition, we have included the sex information for each animal in our open-source code and data. We hope that the availability of this data can help inform researchers developing future experiments, even if statistical comparisons of stimulus response cannot be drawn with our current sample size.

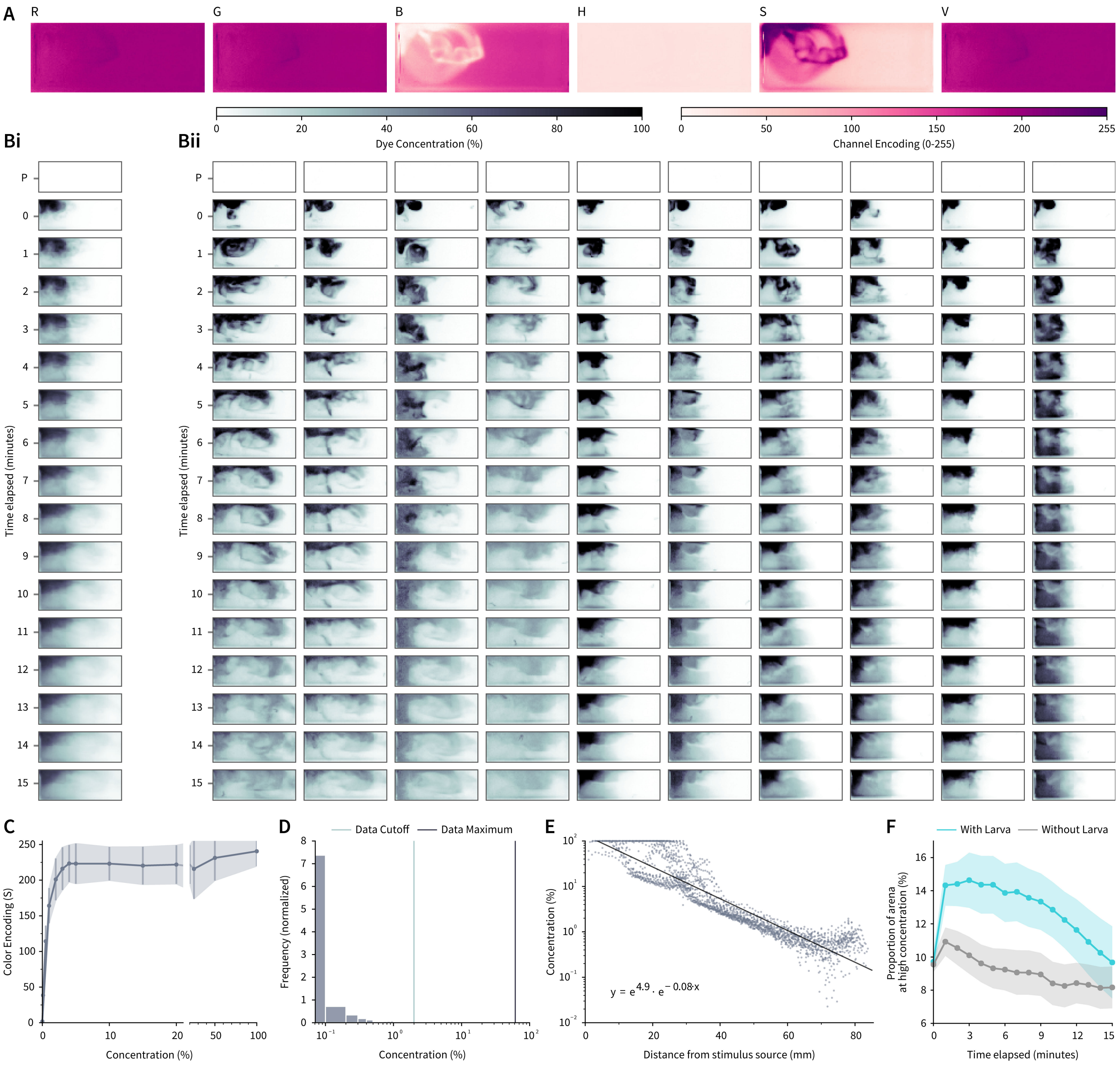

Figure S2: Creating a concentration gradient map to analyze and model larval search behavior. A: To quantify fluorescein dye diffusion, photographs were taken every minute using a Canon PowerShot ELPH 320 HS camera. Of the available color information channels (RGB, HSV), the saturation channel (S) contained the most information and was used to represent dye color throughout image analyses. **Bi:** Dye diffusion through time was quantified by the mean of all values in each 1mm^2 area, linearly interpolated through time (n=10 experiments containing larvae). A control photograph was taken before the start of each experiment (P) but was not used to construct the chemical gradient map. **Bii:** Individual variation between trials. Each column represents data from one experiment through time. **C:** Dye color (S) was converted to raw concentration values using a standardization dataset of 13 reference concentrations. 20mL of each reference concentration was poured into the entire arena and photographed. **D:** Because 100μL of dye is immediately diluted in the 20mL behavior arena water volume, reference concentration colors could not be used to directly convert color to % maximum concentration. Instead, the maximum concentration value was normalized to $\geq$95% of all color measurements across all experiments. **E:** To create a concentration map for computational simulations in different arena sizes, we analyzed the relationship between concentration and distance from stimulus source at time=0. Concentration values for individual $1\text{x}1\text{mm}^2$ sections across all 10 experiments at time=0 (dots), best fit line (black). **F:** Presence of a larva within the container significantly increases the spread of fluorescein dye. As a proxy for dye distribution, we measured the proportion of 1mm^2 segments within the arena with a concentration of >50%. Blue: Proportion of >50% segments in experiments with larvae; Gray: Proportion of >50% segments in experiments without larvae (n=10 each, mean ± standard error). Initial dye distribution (time=0) was not significantly different between treatments, suggesting that subsequent observation differences are not due to experimenter bias in dye addition (p=0.76, Mann-Whitney U test). Linear regression of dye distribution for all subsequent time steps (time>0) showed significant differences between containers with and without larvae ($p<0.0001$).

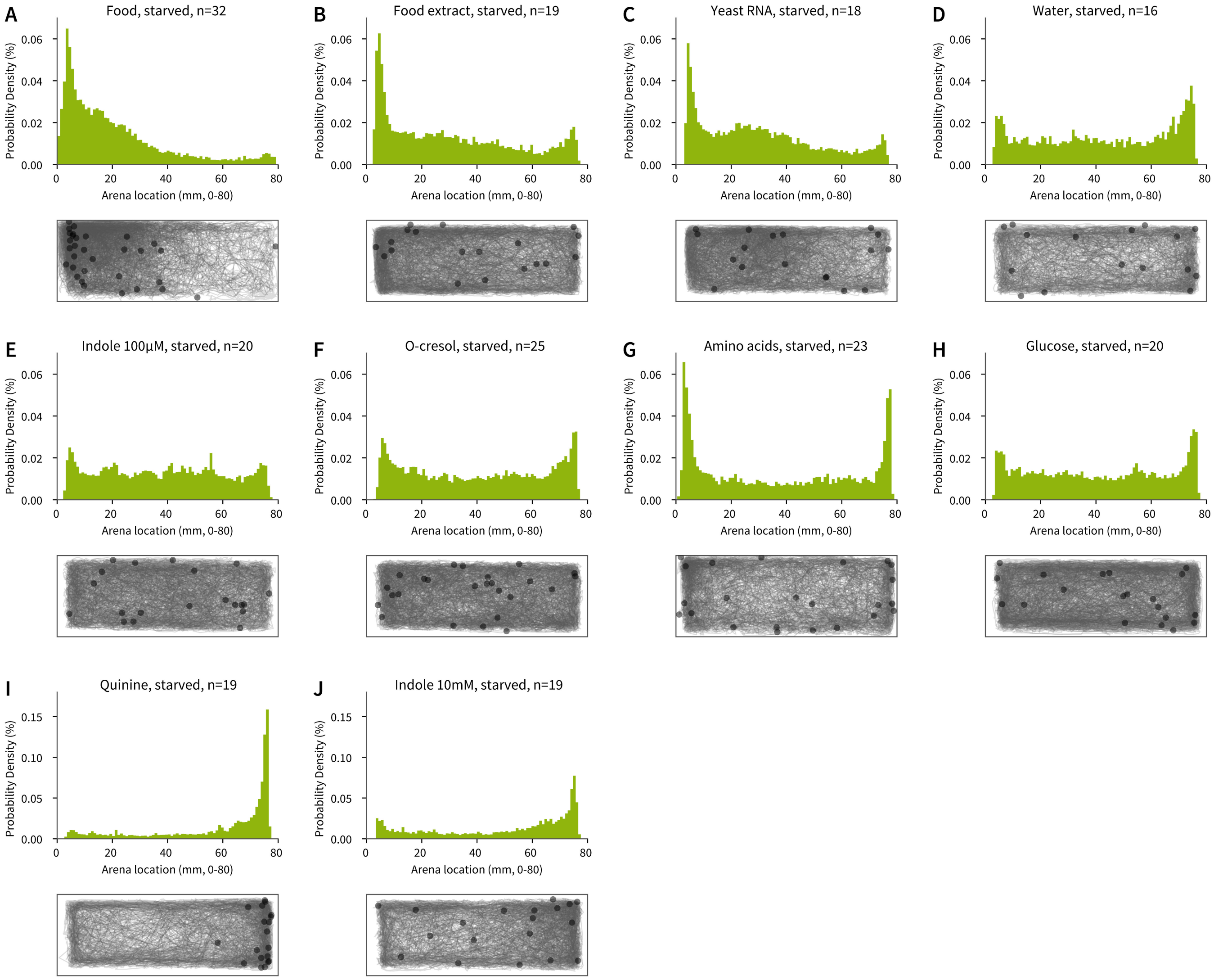

Figure S3: Response of starved larvae to experimental stimuli. A-J: Distribution and trajectories of all starved animals during the experiment phase for food (**A**), food extract (**B**), yeast RNA (**C**), water (**D**), 100μM indole (**E**), o-cresol (**F**), amino acid mixture (**G**), glucose (**H**), quinine (**I**), and 10mM indole (**J**). Although trajectories are shown aggregated into one image for each panel, all animals were tested individually. Scatter points show the position of each animal at the end of the experiment. It is important to note that these histograms show the aggregated position data from all animals throughout the entire 15-minute experiment. Thus, a single animal exhibiting strong attraction or aversion may disproportionately influence this data visualization. For statistical tests reported in this paper, a single preference value was calculated for each animal (Figure 3C) to avoid such effects.

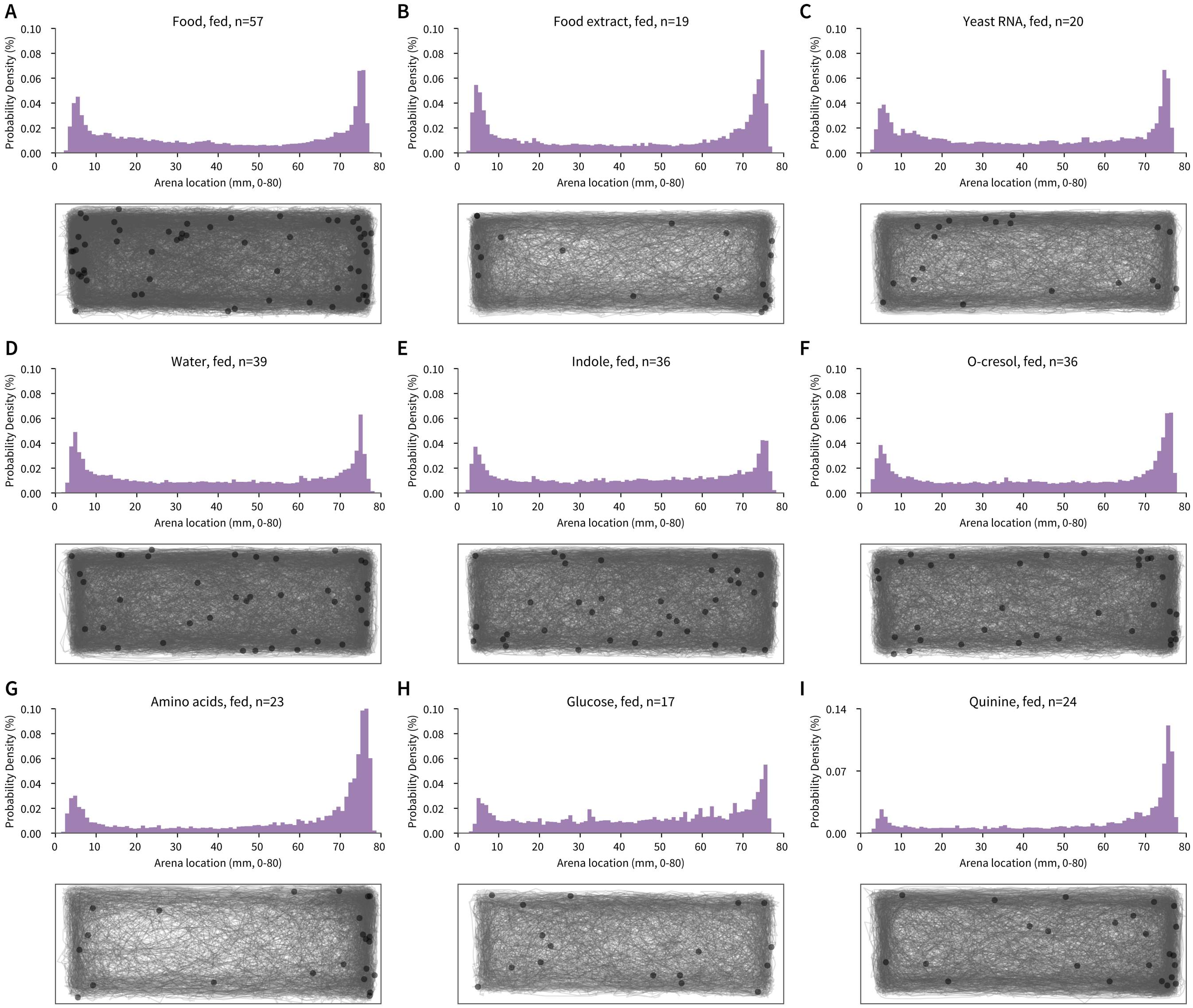

Figure S4: Response of fed larvae to experimental stimuli. A-I: Distribution and trajectories of all starved animals during the experiment phase for food (**A**), food extract (**B**), yeast RNA (**C**), water (**D**), 100μM indole (**E**), o-cresol (**F**), amino acid mixture (**G**), glucose (**H**), and quinine (**I**). Although trajectories are shown aggregated into one image for each panel, all animals were tested individually. Scatter points show the position of each animal at the end of the experiment. Note that the high distribution peaks observed at each side of the arena visualize the higher preference for walls observed in fed animals (Fig 4B). As in Fig S3, it is important to note that these histograms show the aggregated position data from all animals throughout the entire 15-minute experiment. Thus, a single animal exhibiting strong attraction or aversion may disproportionately influence this data visualization. For statistical tests reported in this paper, a single preference value was calculated for each animal (Figure 3C) to avoid such effects.

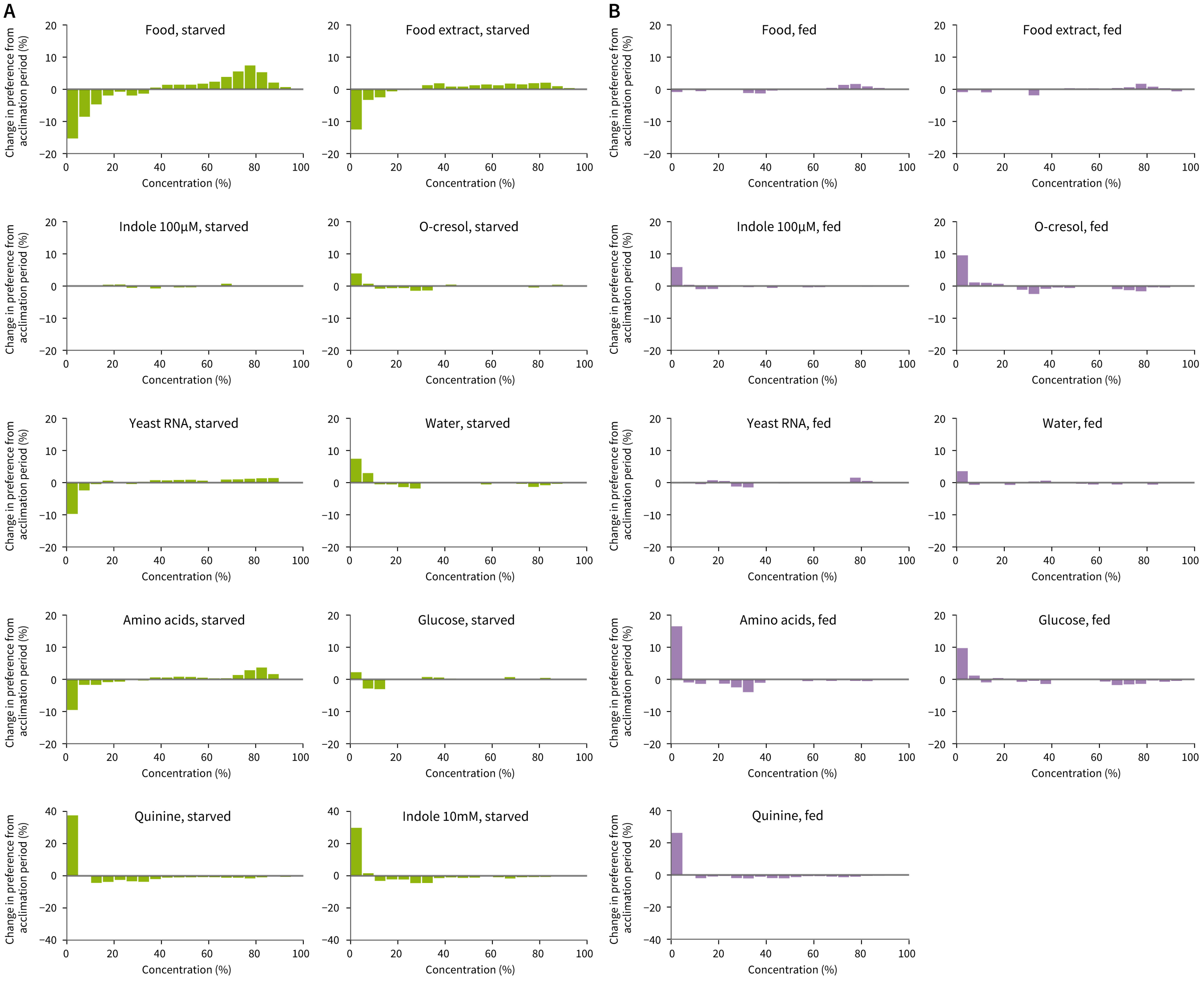

Figure S5: Behaviorally relevant stimuli concentration thresholds for starved and fed larvae. Distribution of starved (**A**) and fed (**B**) animals across the stimulus concentration map. **A, B:** Histograms visualize the change in preference for each concentration bin, normalized to larval distribution during the acclimation phase. This visualization is provided to suggest an estimate for the stimulus concentration thresholds that may be behaviorally relevant for larvae. As in Fig S3 and S4, it is important to note that these histograms show the aggregated position data from all animals throughout the entire 15-minute experiment. Thus, a single animal exhibiting strong attraction or aversion may disproportionately influence this data visualization. For statistical tests reported in this paper, a single preference value was calculated for each animal (Figure 3C) to avoid such effects.

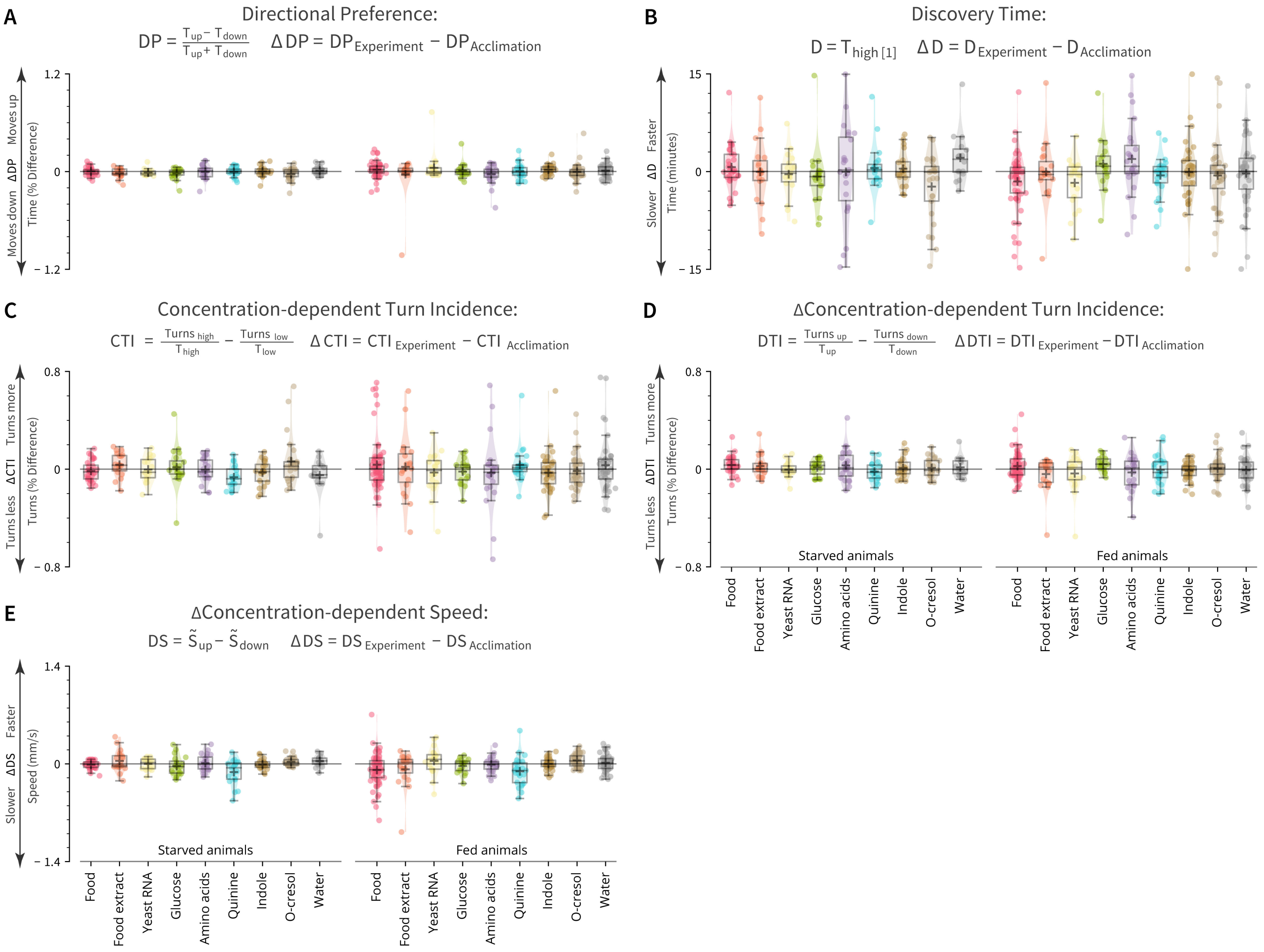

Figure S6: Larval behavior is not consistent with chemotaxis or klinokinesis search strategy models. A-E: Box plots for the population median ± 1 quartile, population mean (+ marker) and mean response for each individual (dots). We observed no significant changes across stimuli for any of these five behavioral metrics ($p>0.05$, Kruskal-Wallis test). Equations above plots denote how the behavioral metrics were calculated. **A:** Directional Preference ΔDP, difference in time (T) moving up or down the concentration map. **B:** Discovery time ΔD, time (T) elapsed before initial encounter of high concentration (≥50%). **C:** Concentration-dependent Turn Incidence ΔCTI, difference in turning rate at high and low local concentrations. **D:** ΔConcentration-dependent Turn Incidence ΔDTI, difference in turning rate while moving up or down concentration. **E:** ΔConcentration-dependent Speed ΔDS, difference in mean speed ($\tilde{S}$) while moving up or down the concentration map.

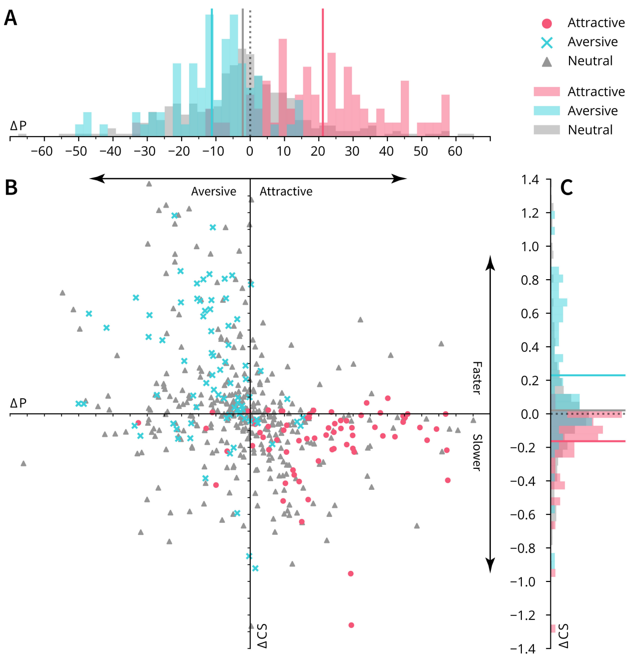

Figure S7: Larval stimulus preference is correlated to concentration-dependent movement speed. A: Normalized frequency histograms of ΔP. Mean response to aversive, neutral, and appetitive cues are visualized as solid vertical lines in the corresponding color. A dotted black line at zero indicates the expected outcome if the added stimulus had no effect on larval behavior. **B:** Larval preference (ΔP) significantly correlates with Concentration-dependent Speed (ΔCS). Results from all experiments are shown grouped into three categories: attractive (pink: food, food extract, and yeast RNA in starved larvae), aversive (blue: quinine in fed and starved larvae; o-cresol in fed larvae), and neutral (grey: water, indole, glucose, and amino acids in fed and starved larvae; o-cresol in starved larvae; food, food extract, and yeast RNA in fed larvae). **C:** As in B, except for normalized frequency histograms of larval ΔCS.

	**Radius**	**Frequency**	**Examples**
**i**	<5cm	27.8% of habitats	Ant traps
**ii**	5-9cm	9.7% of habitats	Tin cans, bottles
**iii**	9-17cm	32.3% of habitats	Jars, bowls, vases
**iv**	17-20cm	3.1% of habitats	Plates, pails

Table S1: Ecologically realistic habitat sizes analyzed through computational modeling. A range of habitat sizes were selected from a literature search of realistic habitat sizes for *Ae. aegypti* larvae ([46] and references therein).

Supplemental Materials and Methods

Insects

Wild-type *Ae. aegypti* (Costa Rica strain MRA-726, MR4, ATCC Manassas Virginia) were maintained in a laboratory colony as previously described [47]. Experiment larvae were separated within 24 hours of hatching and reared at a density of 75 per tray (26x35x4cm). One day before the experiment, 4-day-old larvae were isolated in Falcon^{TM} 50mL conical centrifuge tubes (Thermo Fischer Scientific, Waltham, MA, USA) containing ∼15mL milliQ water. Starved larvae were denied food for at least 24 hours before the experiment. Animals that died before eclosion or pupated during the experiment were omitted. Because it was not possible to detect younger larvae using our video tracking paradigm, we mitigated possible age-related behavioral confounds by standardizing the age of experimental larvae.

Selection and Preparation of Odorants

Odorants (indole, o-cresol) were prepared at 100μM in milliQ water (Aldrich #W259306; Aldrich #44-2361) - a concentration previously shown to be significantly attractive to *An. gambiae* mosquito larvae [23]. Indole was also prepared similarly at 10mM, a concentration that is significantly aversive to *An. gambiae* larvae [23]. Quinine hydrochloride was prepared at 10mM in milliQ water (Aldrich #Q1125). Larval food (Petco; Hikari Tropic First Bites) was prepared at 0.5% by weight in milliQ water and mixed thoroughly before each experiment to resuspend food particles. To prepare the food extract solution, 0.5% food was dissolved in milliQ water for one hour and filtered through a 0.2μm filter (VWR International #28145-477). For the yeast RNA solution, total RNA from *Saccharomyces cerevisiae* yeast was prepared at 0.1% by weight in DEPC-treated, autoclaved 0.2μm filtered water (Aldrich #10109223001; Ambion #AM9916). Yeast RNA, food, and food extract were prepared fresh daily. Glucose and the amino acid mixture were prepared at concentrations previously shown to be optimal for rearing *Ae. aegypti* larvae [27]: D-(+)-Glucose (Aldrich #G8270) was prepared at 10g/L, and the amino acid solution consisted of L-lysine (Aldrich #L5501, 0.66g/L), L-tryptophan (Aldrich #T0254, 0.36g/L), L-histidine (Aldrich #H8000, 0.25g/L), L-leucine (Aldrich #L8000, 1g/L), L-isoleucine (Aldrich #I2752, 1.12g/L), L-threonine (Aldrich #T8625, 0.75g/L), L-methionine (Aldrich #M9625, 0.7g/L), L-valine (Aldrich #V0500, 1.2g/L), and L-arginine (Aldrich #A8094, 0.39g/L). Although chemicals diffuse at different rates depending on molecular size and physico-chemical properties, diffusion coefficients in water were unavailable for the majority of mixtures tested. Therefore, it is important to note that our chemical diffusion map is an approximation of the actual chemosensory environment experienced by larvae. Nonetheless, active behavior of the larva modified the chemical distribution in the arena to such a degree that any differences would be negligible.

Statistical Analyses

Statistical analyses were performed in R version 3.5.1 [48]. A Bonferroni-Holm correction was applied to statistical analyses. A non-parametric Mann-Whitney test was used to compare body length of fed and starved males and females, because a Shapiro-Wilk normality test demonstrated that the data was not normally distributed ($p<0.05$) (Fig S1A). Linear least squares regression was used to assess the effect of time of day to animal speed, time spent moving, and time spent near walls during the acclimation phase (Fig S1B-D). Paired-samples Welch's t-tests were used to compare the median chemical concentration chosen by the larvae throughout the 15-minute experiment to the behavior of the same larvae throughout the 15-minute acclimation phase. This preference metric was also quantified as a single value (ΔP, $P_{Experiment}$-$P_{Acclimation}$, Fig 3, Fig S4). For all subsequent analyses on behavioral mechanisms, larval behavior during the acclimation phase was subtracted from larval activity during the experiment phase to normalize for differences between individuals and larval preference for corners and walls. When investigating potential differences between attraction and aversion behaviors, we grouped stimuli into cues that elicited significant attraction ($\Delta P>0$, $p<0.05$), significant repulsion ($\Delta P<0$, $p<0.05$), or neutral response ($p\geq0.05$). A non-parametric Kruskal-Wallis test was used to compare behavioral metrics among these three stimuli classes, because a Shapiro-Wilk normality test demonstrated that the data was not normally distributed ($p<0.05$) (Fig 3D, Fig S3, Fig S4). These other behavioral metrics included Directional Preference (ΔDP), defined as the difference in time moving up or down the concentration map; Discovery time (ΔD), defined as the time elapsed before initial encounter of high ($\geq$50%) concentration of the stimulus; Concentration-dependent Speed (ΔCS), defined as the difference in speed at high ($\geq$50%) and low (<50%) local concentrations; ΔConcentration-dependent Speed (ΔDS), defined as the difference in speed while moving up or down the concentration map; Concentration-dependent Turn Incidence (ΔCTI), defined as the difference in turning rate (turns per second, turns defined as instantaneous change in angle of >30°) at high and low local concentrations; and ΔConcentration-dependent Turn Incidence (ΔDTI), defined as the difference in turning rate while moving up or down the concentration map. For statistical analyses, larvae that never entered areas of high concentration were assigned a ΔD of 15 minutes, corresponding to the end of the experiment, and a ΔCS and ΔCTI of 0 (placeholder values chosen to reduce Type I error). We did not conduct statistical analyses on simulated data, and instead report overall trends in the results throughout the manuscript. This approach was chosen because the large number of replicates, which were necessary for reducing the noise introduced by randomizing the larval starting location, would artificially inflate the significance of statistical comparisons.
